# Tannery Effluent Treatment by Nanofiltration, Reverse Osmosis and Chitosan Modified Membranes

**DOI:** 10.3390/membranes10120378

**Published:** 2020-11-28

**Authors:** Asmaa Zakmout, Fatma Sadi, Carla A. M. Portugal, João G. Crespo, Svetlozar Velizarov

**Affiliations:** 1 Laboratory of Electrochemistry, Corrosion Metallurgy and Inorganic Chemistry, Faculty of Chemistry, University of Sciences and Technology, Houari Boumediene, USTHB, B.P. 32 El Alia, Bab Ezzouar, Algiers 16111, Algeria; zakmout_a@yahoo.fr (A.Z.); sadifatma4444@yahoo.fr (F.S.); 2 LAQV-REQUIMTE, Department of Chemistry, Faculty of Science and Technology, Universidade Nova de Lisboa, P-2829-516 Caparica, Portugal; jgc@fct.unl.pt

**Keywords:** tannery effluent, chromium recovery, nanofiltration, reverse osmosis, chitosan membranes

## Abstract

The objective of this work is to develop an appropriate technology for environmentally sound membrane-based purification of a tannery effluent assuring, simultaneously, the recovery of chromium, considered as the most hazardous inorganic water pollutant extensively used in leather tanning. A comparison between the permeate fluxes obtained during treatment of a synthetic tannery effluent through nanofiltration (NF270 and NF90 membranes) and reverse osmosis (BW30 and SW30) membranes was first performed. Then, a dedicated polymeric membrane was prepared by coating chitosan (cs) on a polyethersulfone (PES) microfiltration membrane (cs-PES MFO22) support. The resulting membrane was characterized by Fourier Transforms Infrared Spectroscopy Attenuated Total Reflectance (FTIR-ATR), Emission Scanning Electronic Microscopy (SEM) to confirm the process of surface modification and cross-linking of chitosan with glutaraldehyde. This membrane was found to be highly effective for chromium removal (>99%), which was more than eight times higher in reference to monovalent cations (e.g., Na^+^ and K^+^) and more than six times higher in reference to the divalent cations (Mg^2+^ and Ca^2+^) studied. The reverse osmosis permeate conforms to local Algerian regulations regarding being discharged directly into the natural environment (in this case, Reghaia Lake) or into urban sewers linked to wastewater biological treatment stations. While the SW30 membrane proved to be the most effective for purification of the tannery effluent, the chitosan modified membrane proved to be appropriate for recovery of chromium from the reverse osmosis concentrate.

## 1. Introduction

Nowadays, industrial waste is the most common source of water pollution, which continues to increase because most countries are becoming industrialized [1]. Thus, the environment is under increasing pressure from wastes emanating from such industrial activities [2]. The leather tanning industry has been taken into consideration concerning its environmental impact [3,4]. Tanning is the most polluting operation during the leather making process [4,5], because the predominant leather tanning method is based on the use of chromium salts, specifically trivalent chromium salts, such as chromium(III) sulfate, which is the most widely used chemical in tanneries [6,7,8,9]. About 60% of the total amount of chromium is consumed during the tanning process by reaction with animal skin. The remaining chromium rests in the tanning bath and is subsequently discharged into the process wastewater. The toxicological impact of tannery wastewaters containing numerous potentially harmful compounds, including chromium, is very harmful to water bodies and floors [10,11].

The choice of an adequate wastewater treatment process depends on several factors such as efficiency, cost and environmental sustainability. There are several physicochemical methods available for the treatment of tannery wastewater such as: coagulation and flocculation, and biological treatment, but they present relevant shortcomings, such as the excessive production of sludge [8].

In recent years, membrane technologies have been advancing and their costs have been reduced, thus extending the range of their potential applications. Membrane processes have already been used in a large-scale reclamation of tannery wastewater [8,12,13,14,15,16]. The success of applying membranes depends on proper pretreatment of the effluent, and, especially, on the recovery of chromium from the residual waters of leather tanning. Studies applying crossflow microfiltration (MF) [8] and ultrafiltration (UF) [12] have been reported. However, these processes have not been found to be economically viable for this application, because of the porous nature of the membranes which makes them more prone to internal fouling. Therefore, these two processes are feasible only if membrane fouling can be controlled.

A reverse osmosis (RO) system has allowed for the reuse of a purified permeate of a tannery effluent within the production cycle, thus reducing groundwater consumption, which certified the high quality of the permeate produced [8], while UF and MF membranes, due to their lager porous polymeric structures (compared to RO membranes), have allowed higher volumetric permeate fluxes before discharge [13,14].

The objective of recovering chromium led us to the preparation of a new chitosan modified membrane, by coating a chitosan layer on top of a microfiltration polyethersulfone (PES) membrane with an average pore size of 0.22 μm, focusing on its chromium removal capability.

Chitosan is a polysaccharide polymer derived from the second most available natural polymer on earth, chitin, which is found, for example, on the exoskeleton of crustaceans, crabs and shrimp shells [17,18]. Chitosan is one of the best top layer materials and has demonstrated good prospects for use as a raw material for films and membranes preparation [18,19]. Most of the applications of chitosan copolymers employ the use of cross-linkers, such as glutaraldehyde (GDA), in order to improve their stability [20,21,22,23]. Chitosan copolymers represent a versatile biomaterial platform for wastewater treatment applications and the separation of toxic metals due to their hydrophilicity, non-toxicity, adsorptivity, biocompatibility and relative availability and amenability to chemical modification [18,24,25]. Chitosan shows adsorption properties [22,23,24,25,26,27,28,29,30,31] ascribed to the presence of amino and hydroxyl groups, which explain their affinity towards metals—e.g., copper, iron, chromium—and their capacity to coordinate with these species [23,26,27,29,31].

**Scheme 1:**
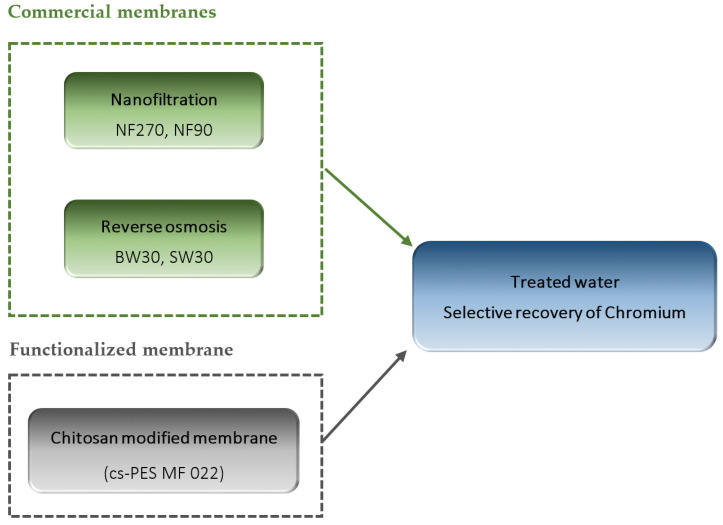
Diagram showing the experimental work plan and final goals.

The main aims of this study are to develop an appropriate membrane-assisted treatment of tannery effluents that simultaneously allows for the selective recovery of the chromium species present. The specific objectives are (i) to identify the optimal conditions to concentrate all existing species present in the tannery effluent, comparing the performance of nanofiltration (NF) and RO membranes, (ii) to obtain a treated effluent with a quality permitting its reuse and/or discharge into the environment, (iii) to modify a polymeric membrane support, using chitosan cross-linked with glutaraldehyde and evaluate the modified membrane performance in terms of its selectivity to preferentially remove chromium-containing species (Scheme 1).

## 2. Experimental

### 2.1. Materials

#### 2.1.1. Chemicals

The reagents used were: magnesium chloride (MgCl_2_; 99%) provided by Alfa-Aesar Kandel, Germany), calcium chloride 2-hydrate (CaCl_2_.2H_2_O; >99%), Ammonium sulphate ((NH_4_)_2_SO_4_; > 99%) and sodium chloride (NaCl; 99.8%) supplied by Applichem PANREAC (Barcelona, Spain), as well as chromium(III) sulphate basic (Cr_4_(SO_4_)_5_OH_2_; 26% Cr_2_O_3_), obtained from Fluka Analytical (Buchs SG Switzerland). Chitosan (cs) was provided by Sigma-Aldrich (St. Louis, MO, USA). Acetic acid (CH_3_COOH; 99.8%), potassium sulphate (K_2_SO_4_; >99%) and sodium hydroxide (NaOH; >93%) were supplied by Carlo Erba (Val de Reuil, France). Glutaraldehyde (GDA; 25% in H_2_O) was purchased from Sigma-Aldrich Chemicals (St. Louis, MO, USA) and hydrochloric acid (HCl; 35–38%) from Sigma-Aldrich Chemicals (St. Louis, MO, USA).

#### 2.1.2. Solutions

##### Tannery Effluent of TAMEG-Rouiba

Samples were taken from the final batch of wastewater discharged from the TAMEG-Rouiba tannery effluent, in order to analyze its composition.

The tannery of TAMEG-Rouiba-SPA, a Leather Industries’ affiliate established in 1966 in its plant of Rouiba, TAMEG Rouiba-Spa, located 25 km east of Algiers, Algeria, in the industrial area of Rouiba, was used. The tannery wastewater of TAMEG-Rouiba is discharged into an industrial sewage system and returned to the environment via the Reghaia Lake. In this case, the effluent treatment standards set by the authorities are governed and imposed on the leather industry to treat the effluent in order to avoid the associated problems [32,33].

The solution temperature and pH were measured in situ. A pre-filtration was performed to the industrial effluent using a membrane filter with an average pore size of 0.45 µm (Gelman Sciences, Portsmouth, UK) before analyses, to remove suspended matter. As can be seen in Table 1 and Table 2, the quality of the TAMEG-Rouiba tannery effluent, regarding most of the parameters, is not acceptable for discharge, according to the provisional standard limits set by the Algerian wastewater quality legislation [34].

##### Synthetic Tannery Effluent

A synthetic solution with the same composition in the inorganic salts as the industrial effluent (Table 3) was prepared by dissolving the required compounds in deionized water, in order to investigate the performance of the membranes under well-defined and reproducible conditions.

#### 2.1.3. Membranes

##### Nanofiltration and Reverse Osmosis Membranes

Five membranes were tested: two commercial nanofiltration membranes-NF270, NF90; two commercial RO membranes-BW30 and SW30 (The Dow Chemical Company, Midland, MI, USA) and a chitosan modified membrane (referred to as cs-PES MF022) prepared by modifying a microfiltration (MF) membrane of polyethersulfone (PES) with an average pore size of 0.22 μm.

The main characteristics of the commercial membranes, based on the information obtained from the manufacturers, are presented in Table 4. All membranes are hydrophilic, as indicated by their reported average water contact angles (θ < 90°), equal to about 29° (for NF270), 51° (for NF90), 55° (for BW30) and 62° (for SW30) [35].

NF270 has a relatively open polymeric structure, reflected in lower NaCl rejection values (Table 4), while NF90 and BW30 are classified as a tight NF and a loose RO membrane, respectively [36]. SW30 is a tight RO membrane, widely used for seawater desalination [37]. In this way, the selected membranes span the range from NF to RO, which may be necessary to obtain sufficient rejection of the target compounds contained in the effluent.

##### Preparation of Chitosan Modified Membranes

Chitosan membranes were prepared by coating a 2.5% (*w/w*) chitosan solution on a polyethersulfone microfiltration membrane (PES MF022, 0.22 µm) from Merck Millipore (Carrigtohill, Ireland) which was used as a support. A 2.5% (*w/w*) chitosan solution was prepared by dissolving chitosan in a 5% (*v/v*) acetic acid solution. This solution was then casted on the top of a PES MF022 support using an Elcometer casting knife film applicator (E.U), by setting an application air gap at 90 μm, in order to assure a chitosan layer with an uniform and reproducible thickness. The resulting membrane was kept and dried at room temperature in a fume hood until total solvent evaporation. The membrane was then soaked in a 1 M NaOH solution for four hours. The membrane was immersed in a glutaraldehyde solution (25% in H_2_O) overnight and then washed exhaustively with distilled water. Membrane washing was followed by measuring the pH, the conductivity and the absorbance spectra (within a λ range of 200–400 nm) of the washing water collected, obtained with a UV–Visible spectrophotometer Thermo Scientific Evolution 2O1 (Madison, WI, USA), in order to check if there is any loss of loosely bound compounds. The washing procedure was complete when pH and conductivity were equal to their values in distilled water and the absorbance was close to zero.

##### Structural and Chemical Characterization of the Chitosan Modified Membrane

The membrane surface was analyzed by Field Emission Electronic Microscopy (FEG-SEM) to assess membrane structure (membrane surface and cross-section) and the chemical characteristics of the membrane surface. The membrane samples were coated with an Au/Pd film of 20 nm thickness, using a sputter coater from Quorum Technologies (model Q150TES, West Sussex, UK) and analyzed in a FEG-SEM system from JEOL (model JSM7001F, Tokyo, Japan).

The membrane chemical structure was also inspected by infrared analysis, using a Fourier Transform Infrared Thermo Scientific Nicolet 6700 FTIR (PerkinElmer®, Waltham, MA, USA) with Attenuated Total Reflectance (ATR) equipment. The software package Omics was used to analyze the signals obtained from the sample.

### 2.2. Methods

#### 2.2.1. Experimental System

All filtration experiments were carried out in a laboratory test unit. A membrane area of 51.4 cm^2^ was used in a dead-end stainless steel test cell—METCell, supplied by MET, UK. In this test cell, the membrane is supported by a porous stainless steel disc. The applied pressure was varied within the range from 20 to 40 bar for NF and RO membranes and from 5 to 40 bar for chitosan modified membranes with a MET pre-assembled nitrogen gas unit. During the experiments, the solutions were stirred at 400 rpm. The membranes’ hydraulic permeabilities were measured throughout the experiments and a new membrane disc was used whenever the membrane hydraulic permeability differed by more than 10% in relation to its original value. Permeate was collected in a recipient vessel during the course of each experiment and the flux was monitored by acquisition of the permeate weight, using an electronic balance with an accuracy of 0.1 g connected to a PC. Pre-compaction was performed for the NF and RO membranes until obtaining constant and stable pure water fluxes for each membrane under study under first 40, then 30 and finally 20 bar of applied pressure, which was achieved in 0.5 to 3 h. Once a membrane under study was compacted, the cell was loaded with 200 mL of synthetic or real effluents and pressurized at 20 bar. Each batch permeation filtration experiment was run until achieving an 8-fold reduction in the initial volume in order to allow for a sufficient retentate volume (25 mL) for performing the required physicochemical analyses. Then, the experiment was stopped. The remaining retentate was stored for analyses. Distilled water was finally used to assess the degree of restoration of the pure water permeability of the NF and RO membranes after each filtration experiment.

#### 2.2.2. Data Analysis

The permeate flux in L/(m^2^ h) was calculated as in [38]:

(1) $$J=\frac{Q_{P}}{A}$$

where $Q_{P}$ is the permeate flow (L/h) and *A* is the surface area of the membrane (m^2^).

The membrane hydraulic permeability was calculated by measuring the permeate flux as a function of time and applying the Darcy equation:

(2) $$J=L_{P}*TPM$$

(3) TMP=ΔP−Δπ

where *Lp* is the membrane hydraulic permeability, *TMP* is the transmembrane pressure, ΔP is the applied pressure difference and Δπ *is* the osmotic pressure difference between the retentate and the permeate side (bulk solutions).

In dilute solutions, such as the one used in the present study (all compounds present in the ppm concentration range (Table 3), the van’t Hoff equation can be applied to calculate the osmotic pressure difference between the retentate and the permeate:

(4) $$\Delta \pi =RT\left(iC_{F}-i^{\prime }C_{P}\right)$$

where *R* is the gas constant and *T* is the absolute temperature. $C_{F}$ is the total molar feed concentration, $C_{P}$ is the total molar permeate concentration and *i*, *i′* are the van’t Hoff factors of the feed and the permeate, respectively.

To calculate the osmotic pressure, chemical equilibrium diagrams were obtained using Make Equilibrium Diagrams Using Sophisticated Algorithms (MEDUSA) software (version: Eq.calcs_32) [39].

The osmotic pressures of the feed and final retentate solutions, as well as of all withdrawn permeate solutions at the respective sampling times, were estimated based on their chemical compositions assessed analytically. The osmotic pressure changes in the retentate were estimated through the respective instantaneous mass balances at the specific sampling times, considering negligible amounts of mass accumulated in the membranes.

The volume concentration factor (*VCF*) was calculated as the ratio of initial volume of feed divided by the retentate volume:

(5) $$VCF=\frac{Volume_{initial}}{Volume_{retentate}}$$

The contaminant removal (or apparent rejection (R)) was calculated as:

(6) $$R=\frac{C_{F}-C_{P}}{C_{F}}\times 100\%$$

The selectivity (*S*) was defined as in [40]:

(7) $$S_{A/B}=\frac{R_{A}}{R_{B}}$$

where *R_A_* and *R_B_* are the apparent rejections of chemical compounds *A* and *B*, respectively.

#### 2.2.3. Analytical Methods

An inductively coupled plasma atomic emission spectrophotometer (ICP-AES, (Ultima model, Horiba Jobin-Yvon, France) equipped with a radiofrequency (RF) generator of 40.68 MHz, a Czerny-Turner type monochromator with 1.00 m (sequential), an AS500 autosampler and data acquisition software was employed to determine the concentration of Ca, S, Mg, Na, K and Cr. The pH and conductivity of feed, retentate and permeate solutions were determined using a pH-meter, type CRISON (Barcelona, Spain) and a Schott Lab960 conductivity meter (Mainz, Germany), respectively.

Ammonium content was determined colorimetrically using a Skalar SAN^++^ segmented flow analyzer (Skalar Analytical B.V., AA Breda, The Netherlands) [41]. A total organic carbon (TOC) analyzer (SHIMADZU, Kyoto, Japan) was used to measure the TOC content. Kits (LCK11) from Hach Lange GmbH, Düsseldorf, Germany were used for chloride determination. Chemical Oxygen Demand (COD) was assessed according to the AFNOR XPT 9O-319 protocol in a thermo-reactor Spectro TR 320 by a KIT LOVIBOND; Biological oxygen demand in 5 days at 20 °C (BOD_5_) was determined using the AFNOR NF 1899-2 protocol; Turbidity (Turb.) was obtained directly in Nephelometric Turbidity Units (NTUs) Z using a field turbidimeter 2000 NTU, according to the NF EN ISO 7027, 2000 standard. The TSS content was measured using the AFNOR 38 protocol [42].

## 3. Results and Discussion

### 3.1. Treatment of Synthetic Tannery Effluent with Commercial NF and RO Membranes

#### 3.1.1. Volumetric Permeate Fluxes and Membranes’ Hydraulic Permeability

**Figure 1:**
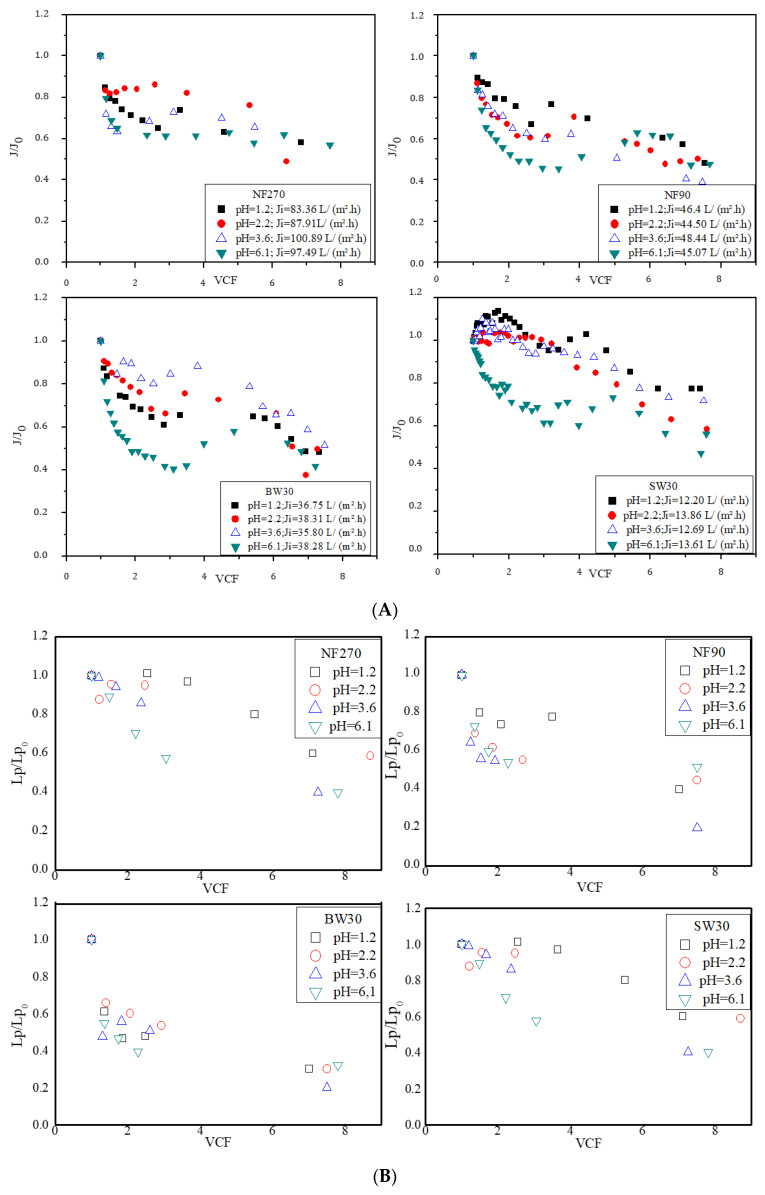
Evolvement of normalized permeate fluxes (**A**) and hydraulic permeabilities (**B**) versus volume concentration factors (VCFs) of a synthetic tannery effluent treated by NF and RO membranes at different pH values.

The effect of pH on membrane performance was evaluated based on the analysis of the normalized (to the initial ones) volumetric permeate fluxes, J/J_0_, and permeabilities Lp/Lp_0_ (Figure 1) at pH values ranging from 2 to 6 (in order to facilitate the comparison between the studied membranes).

For the membranes studied, the drop in the normalized flux was higher at pH 6.1, which can be associated with the presence of membrane scaling due to the deposition/accumulation of inorganic solutes at the membrane surface. Membrane scaling was confirmed by the visual observation of precipitates on the surface of the membranes after operation. The normalized flux decline at pH 6.1 was more severe for the RO membranes, especially for BW30, while it was attenuated for the NF membranes, particularly for NF270, which possesses the most open polymeric structure (allowing for the highest volumetric permeate fluxes and therefore a lower accumulation of deposited materials on its surface). In any case, these deposits were minimal and easily removable by water.

For pH values below 3.6, a slight initial increase in the normalized permeate flux was observed (Figure 1A) for the SW30 membranes. In this pH range, which is slightly below the isoelectric point of this membrane [43], the protonation of carboxylic groups present in the interfacial polymeric layer is expected to lead to a neutral or slightly positive surface charge. Such a slight charge close to the isoelectric point of the membrane surface of RO membranes might be attributed to conformational changes of the cross-linked membrane polymer structure and/or increased water permeability due to a decrease in electro-viscous effects [44].

The normalized flux decline behavior for SW30 is due to both a drop in permeability (see Figure 1B) and an increase in the osmotic pressure difference (associated with the increase in salt concentration in the retentate during the process) leading to a decrease in the useful TMP from 18.9 bar in the beginning of operation, down to 13.5 bar at the end of permeation. This trend is maintained for all membranes. As expected, the decrease in TMP is the smallest for the NF270 membrane, for which the normalized flux decline is mostly due to a decrease in permeability.

The degree of recovery of pure water permeability of the investigated membranes after the synthetic effluent filtration tests was evaluated and compared. The data obtained are presented in Table 5. The highest recovery of 98.99% of the hydraulic permeability was observed for NF270. This result can be associated with the more open polymeric structure of NF270, resulting in an easier washing out of the retained deposited salts. The hydraulic permeability of NF90 decreased from 7.172 L/(h m^2^ bar) before permeation of the synthetic effluent to 0.84 L/(h m^2^ bar) at the end of the process carried out at pH = 2.2, which may be due to a more severe internal fouling. At higher pH values, the recovery of the pure water permeability of BW30 remained practically unchanged (~77%) at pH 3.6 and 6.1.

For SW30, however, the recovery of the pure water permeability increased at a pH of 6.1, which could possibly be related to easier removal of the precipitants formed on its surface during washing, since SW30 has the densest and most smooth top layer surface [43] among the investigated membranes.

#### 3.1.2. Rejection Performance

**Figure 2:**
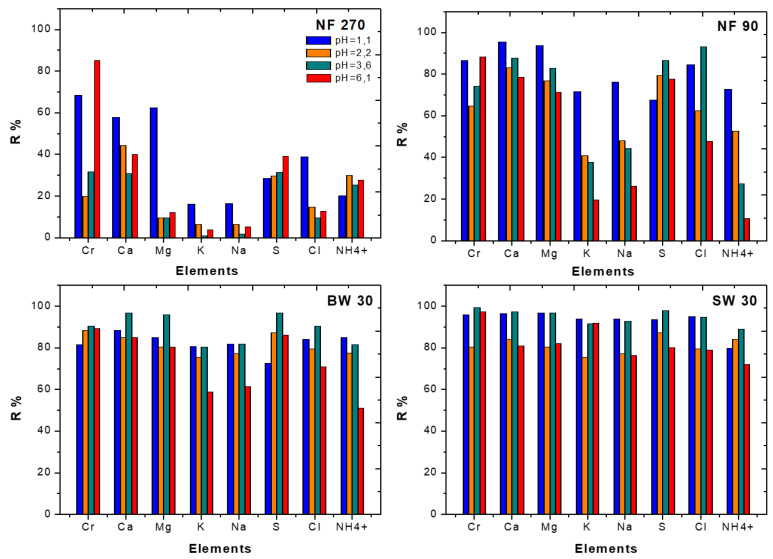
Apparent rejections of the target compounds for the studied RO and NF membranes: **NF270**, **NF90**, **BW30** and **SW30**, respectively.

The apparent rejections of each compound (i.e., Cr, Ca, Mg, K, Na, S, Cl^−^ and NH_4_^+^) present in the synthetic tannery effluents at a VCF of 8 in NF270, NF90, BW30 and SW30 membranes are presented in Figure 2. As expected, the lowest rejections were obtained with the NF270 membranes, progressively increasing for the membranes NF90, BW30 and SW30 in this order. As shown, the ions Na^+^ and K^+^ were the least retained by the membranes, depicting apparent rejection values inferior to 20% with NF270 while reaching 90% with SW30 membranes. The lowest rejections may be attributable to the smaller sizes of these ions. The element highly rejected by NF270 membranes was Cr, with rejection values exceeding 65% at pH 1.2 and 85% at pH 6.1. Although the apparent rejection values varied with pH, the reason of this dependency is not clearly perceived based on the values obtained for the different ions with membrane NF270. The apparent rejection of Na^+^ and K^+^ seems to decrease as the pH varied from 1.2 to 3.5. The same tendency was observed for the NF90 membrane where the apparent rejections of Na^+^, K^+^ and NH_4_^+^ (the least rejected species with NF90) reached 70% at pH 1.2, decaying to values lower than 30% at pH 6, suggesting an effect of the positive charge in the rejection of cations.

The apparent rejections for the RO membranes BW30 and, especially, SW30, become much less dependent on pH, which can be attributed to the dominant contribution of steric effects compared to charge exclusion ones. SW30, being the densest membrane, provides the highest rejections (more than 90% for all studied species). The apparent rejection of the most toxic compound—Cr—reaches 99.6% with the SW30 membrane, whereas values inferior to 90% were obtained with any other membrane at any pH value.

Overall, the rejection data obtained with the NF membranes show that they are not appropriate for Cr recovery since the chromium rejection degrees are not sufficient to obtain permeates free of chromium, which can be directly discharged into the surrounding environment of the tannery plant. On the other hand, the SW30 membrane at pH = 3.6 and 1.2 is appropriate to treat the tannery effluent allowing for a direct discharge of the permeate effluent, according to the Algerian legislation [29], into the natural environment via Reghaia Lake.

The data obtained with the synthetic tannery effluent suggest that the membrane which presents the best potential in terms of providing adequate effluent permeate quality and offering the possibility for chromium recovery is clearly the SW30 membrane at pH = 3.6.

### 3.2. Treatment of Real TAMEG-Rouiba Tannery Effluent with SW30 Membrane

#### 3.2.1. Volumetric Flux and Membrane Hydraulic Permeability

**Figure 3:**
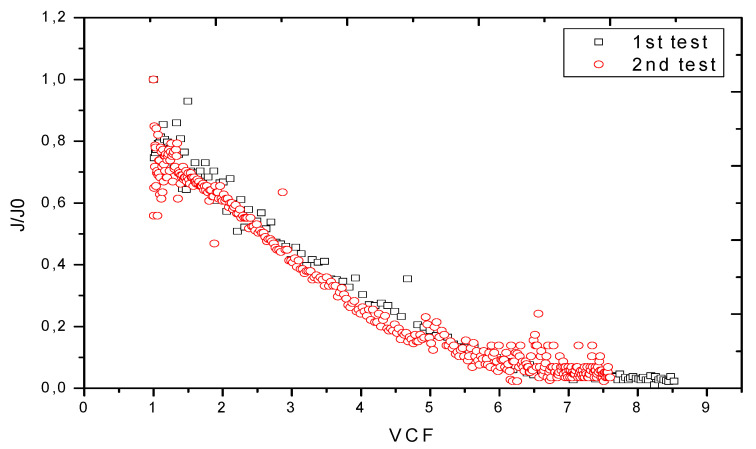
Evolvement of normalized flux profiles of a TAMEG-Rouiba effluent treated by reverse osmosis with an SW30 membrane.

**Figure 4:**
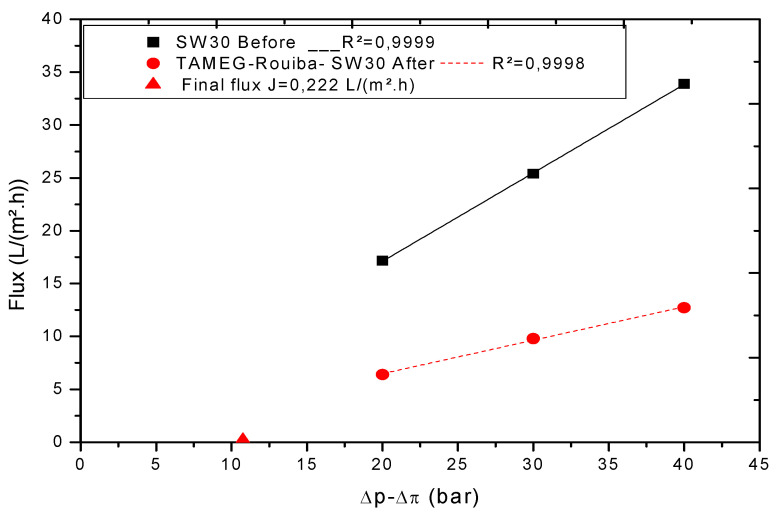
Volumetric permeate flux as a function of transmembrane pressure (TMP).

The application of the process to the real effluent was repeated two times to check the reproducibility. Regarding the evolvement of normalized flux profiles of the TAMEG-Rouiba effluent treated by the SW30 membrane (Figure 3), there is a sharp decline of fluxes between 1.5 ≤ VCF ≤ 6, which is associated with fouling and reduction in the useful TMP, due to increasing osmotic pressure in the retentate. Beyond VCF equal to 6, the flux stabilized. This flux behavior is attributed to the high rejection of the SW30 membrane and the decrease in TMP to 10.75 bar at the end of the process. The permeability (Figure 4) of the SW30 membrane, when the TAMEG-Rouiba effluent is used as feed, is equal to 0.316 L/(h m^2^ bar). This may be due to the existence of organic compounds. The pre-filtration of the industrial effluent has no significant influence on the flux behavior, but the biggest disadvantage of taking the effluent directly may be clogging and non-reuse of the membrane in addition to the long time of permeation due to the concentration of species and the accumulation of organic compounds in the feed near to the membrane surface, which may end up in a reduction in membrane rejection due to an increase in the local concentration of species at the membrane surface.

#### 3.2.2. Rejection Performance

**Figure 5:**
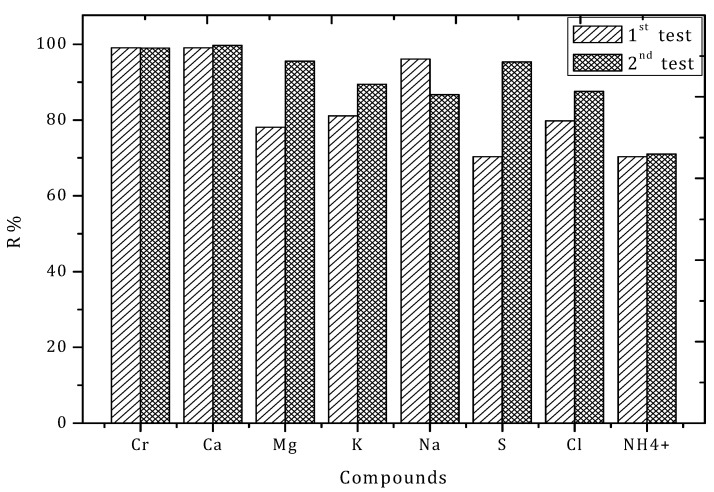
Apparent rejections for the target compounds obtained by filtration of real tannery wastewater across SW30 membrane.

The rejection of each specific element (Cr, Ca, Mg, K, Na, S, Cl and NH_3_) by the SW30 membrane is shown in Figure 5. It is observed that reverse osmosis is effective to concentrate all existing species present in the industrial tannery effluent (>70%) and the results obtained are highly reproducible. These results show that the SW30 membrane may be recommended for the treatment of the tannery effluent in order to obtain a treated effluent, which can be discharged in a natural medium directly, respecting the norm No. 06-141 of 20 Rabie El Aouel 1427, corresponding to 19 April 2006, defining the limit values for discharge of liquid industrial effluents [34].

### 3.3. Treatment of the Synthetic Tannery Effluent with the Chitosan Modified Membranes

The first goal of the work was accomplished by filtration of the tannery wastewater using an SW30 membrane, which led to the production of a treated effluent with reduced concentrations of chemical species, thus allowing its discharge in natural water streams. However, this membrane showed poor selectivity (all elements are identically rejected by SW30 membrane) disabling the selective recovery of Cr as required for the re-use of this chemical element. Therefore, in order to address this demand, chitosan modified membranes (cs-PES MF022) were developed by modification of commercial PES microfiltration membranes (0.22 μm) with chitosan aiming to allow for a selective removal of Cr, taking advantage of the high affinity of chitosan to this element [23,29,31].

The filtration experiments were performed using the synthetic tannery effluent at pH 3.6, in order to favor the removal of Cr(III) species present in solution. Cr(III) is mainly present in aqueous solution in the form of cationic species, such as Cr(OH)_2_^+^ and Cr(OH)^2+^, starting to precipitate at pH values above 3.8 [31]. Therefore, pH 3.6 was selected first to avoid Cr(III) precipitation. The removal of Cr(III) by chitosan modified membranes relies on electrostatic interactions between the cationic Cr species and the amine and hydroxyl groups of chitosan. Amine and hydroxyl groups are predominantly protonated at pH 3.6 (R–NH_3_^+^ and R–OH_2_^+^), thus promoting the rejection of Cr by electrostatic repulsions. Cr removal by electrostatic repulsions to chitosan is expected to decrease as the pH increases, due to the deprotonation of R–NH_3_^+^ and R–OH_2_^+^. However, Cr removal is expected due to the affinity of chitosan to Cr(III), which is explained by the ability of the amine groups of chitosan to coordinate Cr(III). In contrast to the electrostatic repulsions, which should predominate at lower pH values, the coordination of Cr to amine groups are expected to be favored by deprotonation of NH_3_^+^ (pKa = 6.3), which increases with the increase in pH [31].

#### 3.3.1. Structural and Chemical Characterization of the Chitosan Modified Membranes

##### Cross-Linking of the Chitosan Layer

**Figure 6:**
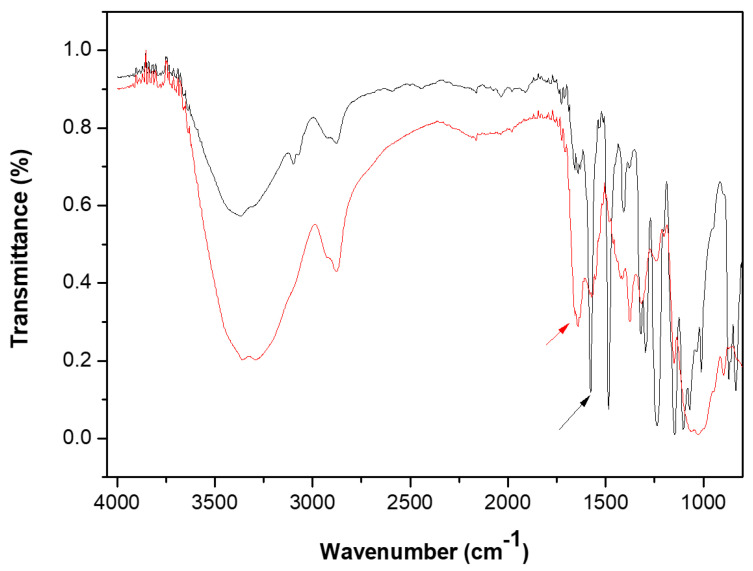
Fourier Transforms Infrared Spectroscopy Attenuated Total Reflectance (FTIR-ATR) spectra of the chitosan modified membranes before and after cross-linking. Black line: chitosan modified membrane before cross-linking; red line: chitosan modified membrane after cross-linking.

Polyethersulfone microfiltration membranes (PES MF022, 0.22 µm) were modified by casting of a chitosan layer on the membrane surface. The stability of the chitosan layer was achieved by cross-linking with glutaraldehyde (GDA), involving the reaction of GDA with the amino groups (NH_2_) of chitosan, resulting in the formation of imine bounds (C=N). Chitosan cross-linking was evaluated by analysis of the Fourier Transforms Infrared Spectroscopy Attenuated Total Reflectance (FTIR-ATR) spectra obtained for the polyethersulfone microfiltration membrane before and after cross-linking, as shown in Figure 6. The presence of cross-linking was confirmed by the increase in the characteristic signal of C=N in the region of 1680–1620 cm^−1^ and the NH_2_ stretching band at 1586 cm^−1^ is attributed to deprotonation of the ammonium cation and cross-linking with glutaraldehyde by comparison with the FTIR-ATR spectra of chitosan [21,22].

##### Stability of the Chitosan Modified Membranes

The chitosan modified membranes were washed by immersion in distilled water through several washing cycles of 1 h each, for removal of loosely bound material until the membrane was stable. The membrane stability was elicited based on changes of pH, conductivity and the absorbance spectra of the supernatant collected after each washing cycle.

**Figure 7:**
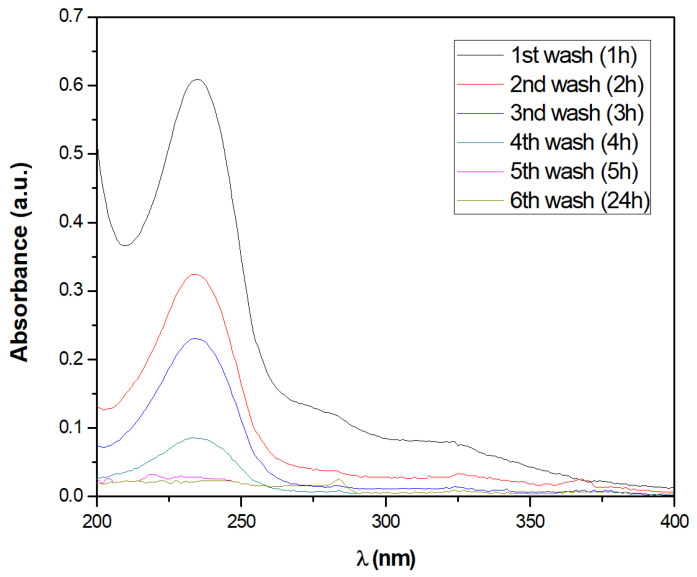
Evolution of absorbance spectra of the supernatant (washing solution) collected during the chitosan (cs)-polyethersulfone (PES) MF022 membrane washing process.

Analysis of the absorbance spectra (Figure 7) revealed the presence of an absorbance band with a maximum at 235 nm, possibly attributable to the release of loosely bound compounds—e.g., chitosan and GDA—from the membrane during washing, which showed a decreasing intensity with the increase in the number of washing cycles. This absorbance band progressively decreased during membrane washing and totally disappeared upon 5 h of washing, corresponding to the time needed to ensure total removal of unbound material. Washing was conducted for a total of 24 h without detection of further UV-Vis signals during this additional period, suggesting the stability of the membrane. The membrane washing efficiency was also monitored based on pH and conductivity of the supernatant collected after each washing cycle.

**Figure 8:**
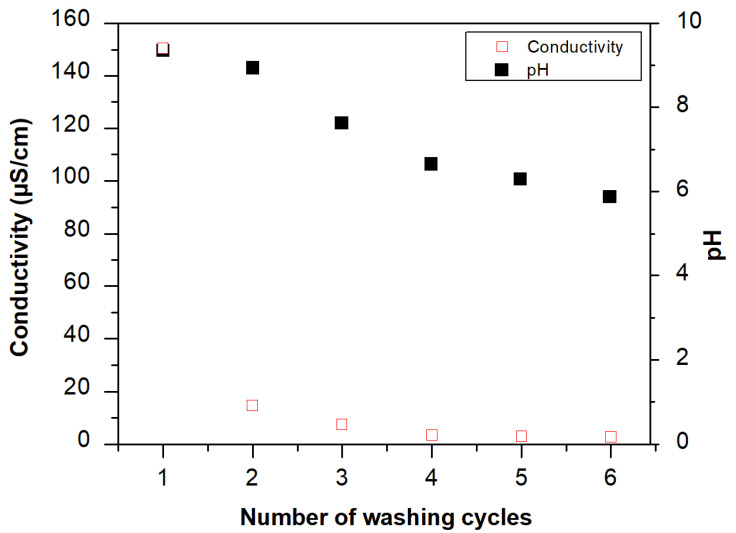
Evolution of conductivity and pH of the supernatant (washing solution) collected during the cs-PES MF022 membrane washing process.

As shown in Figure 8, a decrease in pH from 9 to 5.7 and a decrease in the conductivity from 150.4 to 2.7 μS/cm was observed during the washing cycles. The final pH and conductivity values obtained correspond to the values of the distilled water, revealing a complete membrane washing.

##### Structural Analysis of the Chitosan Modified Membranes

**Figure 9:**
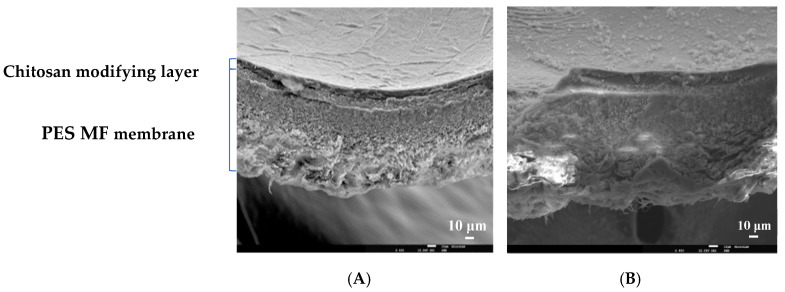
Scanning Electronic Microscopy (SEM) images of the cross-section of the chitosan modified membranes, cs-PES MFO22, obtained (**A**) before and (**B**) after filtration of the synthetic tannery effluent.

Analyses of the cross-section of chitosan modified membranes were investigated by Scanning Electronic Microscopy (SEM). The SEM images obtained (Figure 9), showed the presence of a dense chitosan top layer with ca. 10 μm with good adherence to the polyethersulfone microporous supportThe SEM images did not evidence the physical entrapment of the chitosan inside the membrane pores. Moreover, comparative analysis of the SEM images acquired before and after filtration did not unveil signs of structural disintegration of the chitosan layer, suggesting that the stability of the chitosan modified membrane under the processing conditions.

#### 3.3.2. Treatment of the Synthetic Tannery Effluent with the Chitosan Modified Membrane

##### Permeate Flux and Membrane Hydraulic Permeability

The chitosan modified membrane showed a hydraulic permeability of 0.46 ± 0.03 L/(h m^2^ bar), which is in the same order of magnitude of the value obtained with the reverse osmosis SW30 membrane, at the same pH of 3.6. The low hydraulic permeability exhibited by the chitosan modified membrane is explained by the formation of a chitosan dense layer at the surface, as confirmed by SEM analysis (Figure 9).

The chitosan modified membranes were used for treatment of the synthetic tannery effluents (pH = 3.6) aiming to compare the filtration performance of this membrane with that obtained for the SW30 RO membrane.

**Figure 10:**
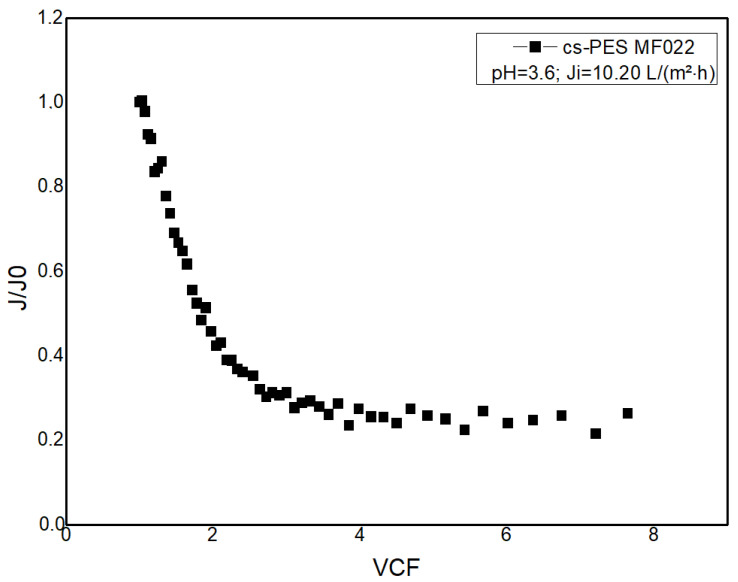
Normalized flux versus volume concentration factor (VCF) using the chitosan modified membrane cs-PES MF 022.

In contrast to the flux profiles obtained with SW30, at pH 3.6, which show a progressive decrease in the normalized permeate flux as the VCF increases, the normalized permeate flux profile obtained with chitosan modified membranes (Figure 10) shows a sharp decline of permeate flux in the initial stage of operation—i.e., for VCF values lower than 3. This permeate flux decline was attributable to concentration polarization and/or fouling effects caused by the concentration build-up of the rejected molecular species at the membrane boundary layer. Since Cr(III) is present in aqueous solutions at low pH values in the form of cationic species, membrane fouling by Cr-containing compounds is very unlikely as electrostatic repulsions should be prevalent. Additionally, and as reported in [31], as precipitation of Cr(III) is only expected at pH values higher than 3.8, membrane scaling due to accumulation of Cr-containing precipitates is not very plausible. However, a possibility of fouling or scaling events caused by interaction of other molecular species with chitosan or precipitation of other metals present in solution at the membrane surface cannot be excluded. Additionally, the contribution of the increase in osmotic pressure in the retentate with the reduction in the useful TMP should not be discarded. This stage was followed by a stabilization of the permeate flux at VCF values higher than 4.

The decrease in the normalized flux profile corresponds to a decrease in the hydraulic permeability of the chitosan modified membranes to 0.25 ± 0.03 L/(h m^2^ bar) after the permeation of the synthetic tannery effluent. These values represent a decrease of 54% in the hydraulic permeability of chitosan modified membranes, which was higher than the decrease of 40% obtained with SW30 membranes.

##### Rejection Performance and Membrane Selectivity

The membrane rejection performance and selectivity of chromium rejection (defined as the ratio of Cr rejection to that observed for other compounds present in the synthetic tannery effluent) was evaluated and compared to the performance obtained with the SW30 membrane.

**Figure 11:**
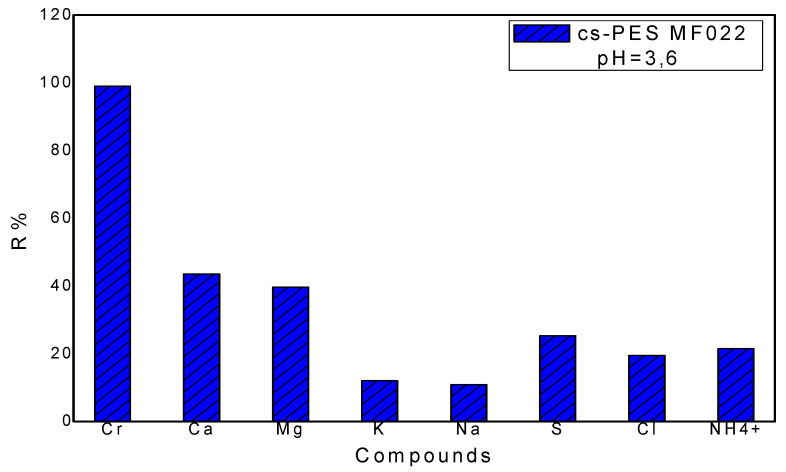
Rejection of the analysed compounds during filtration of synthetic tannery wastewaters at pH 3.6, using the chitosan modified membrane (cs-PES MF 022).

The rejection (R, %) of the different ionic elements with the chitosan modified membrane, represented in Figure 11, clearly showed that the rejection performance of this membrane is significantly lower for most elements than that obtained with the SW30 membrane. The rejection of the chitosan modified membrane was poorer for monovalent elements—R%(NH_4_^+^) = 21.54%, R%(Cl^−^) = 19.45%, R%(K^+^) = 12.06% and R%(Na^+^) = 10.87%—followed by divalent elements—R%(Ca) = 43.52%, R%(Mg) = 39.69%, R%(S) = 25.35%. However, a high rejection of 99.03%, close to that obtained for the SW30 membrane, was found for chromium. The high rejection selectivity for Cr of Cr by the chitosan modified membranes may be explained by the repulsive interactions of the cationic Cr-containing species with the amine and hydroxyl groups which are positively charged at pH 3.6. Additionally, the rejection of Cr by chitosan may be attributed to the coordination of Cr(III) (hard Lewis acid) to the amine group of chitosan (hard base).

**Figure 12:**
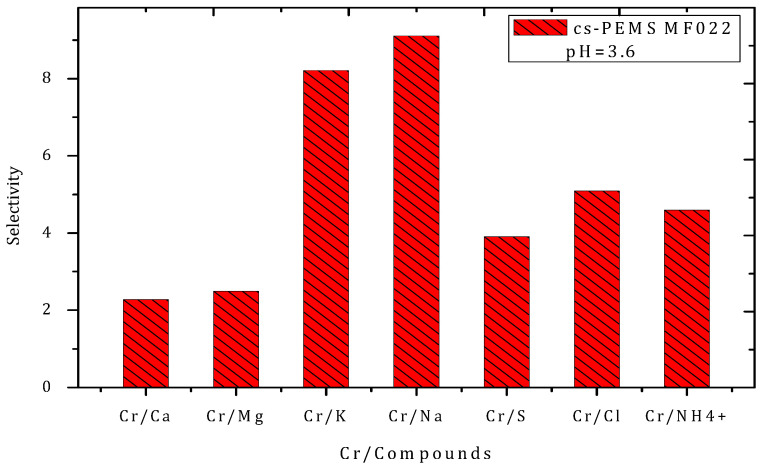
Selectivity of chitosan modified membrane (cs-PES MF022) to chromium relative to the other elements obtained during filtration of synthetic tannery wastewaters at pH 3.6.

Analysis of the rejection selectivity of chromium compared to that of the other studied compounds (Figure 12) shows that the chitosan membrane rejects chromium with a selectivity >8, in case of monovalent cations (Na^+^ and K^+^), with a selectivity between 4 and 6 for anions (Cl^−^ and SO_4_^2−^) and NH_4_^+^, and a selectivity of ~2 in reference to the divalent cations (Mg^2+^ and Ca^2+^). The high rejection selectivity for Cr may be explained by the ability of chitosan to coordinate heavy metals, such as Cr, allied with the inability of this polymer to form complexes with alkali and alkali earth metals, such as Na, K and Mg, as they do not have *d* and *f* unsaturated orbitals [31].

**Scheme 2:**
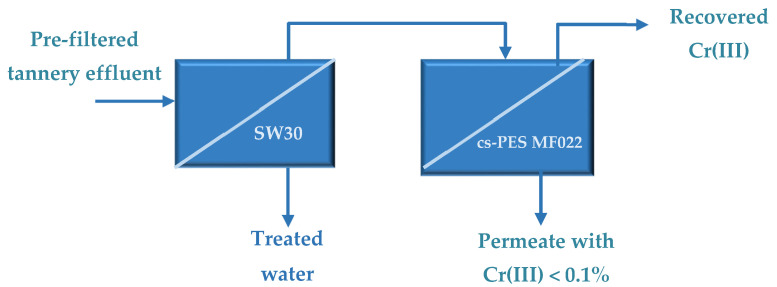
Flowchart of the proposed process for treatment of tannery effluent considering a 1st water purification step using an SW30 membrane and a 2nd process step for selective recovery of Cr(III) using a chitosan modified membrane (cs—PES MF 022).

These results show that the chitosan modified membrane cannot replace the SW30 membrane for purifying the tannery effluent. However, its high rejection selectivity to chromium suggests the possible use of this membrane in a subsequent processing stage, aiming at a selective recovery of chromium retained in the RO concentrate obtained using the SW30 membrane, as illustrated in the flow chart shown as Scheme 2.

## 4. Conclusions

This work compares the performance of different nanofiltration (NF 270 and NF90) and reverse osmosis (BW30 and SW30) membranes in terms of their rejection performance and selectivity when treating a synthetic tannery effluent, which emulates the inorganic content of a real tannery effluent in Algeria. Both BW30 and SW30 membranes lead to a permeate which fulfills the composition criteria imposed by the official journal of the Algerian Republic N° 26, allowing the environmental discharge of this permeate stream. The performance of a chitosan modified membrane prepared by coating with a chitosan layer at the surface of a PES microporous support was also evaluated. Despite their lower permeate fluxes, chitosan modified membranes showed low rejections to monovalent and divalent ionic species making them unsuitable for treating of tannery effluents.

However, the chitosan modified membranes show a selective rejection of Cr, expressed by a Cr rejection higher than 90%, suggesting their potential application for a selective recovery of Cr from tannery effluents in a subsequent processing stage. A potential solution for the tannery effluent involves a first step of reverse osmosis, using an SW30 membrane, which allows one to obtain a permeate that can be disposed and a concentrate enriched in chromium (valuable stream for reuse) that can be further processed by the chitosan modified membrane. The permeate from chitosan modified membranes may then be reintroduced in the process at the inlet of the reverse osmosis step.

Further studies are required for evaluation of the process operaton performance and stability of the modified chitosan membranes during longer-term exposure to the reverse osmosis unit concentrate.

## Figures and Tables

**Table 1:** Physicochemical characteristics of TAMEG-Rouiba Tannery effluent.

Parameter	Temperature	Total Suspended Solids (TSS)	pH	Conductivity	Turbidity	^2^ COD	^3^ BOD
Unit	°C	g/L	Sorensen scale	mS/cm	^1^ NTU	mg O_2_/L	mg O_2_/L
Value	20	1.79	7.1	9.6	322	942	92
Max. levels allowed	30	0.04	6.5–8.5	-	-	130	40

^1^ NTU—Nephelometric Turbidity Units; ^2^ COD—Chemical Oxygen Demand; ^3^ BOD—Biological Oxygen Demand.

**Table 2:** Chemical composition of the TAMEG-Rouiba Tannery effluent after pre-filtration using a membrane filter with an average pore size of 0.45 µm.

Parameter	^1^ COD	^2^ BOD	Cr	Fe	Ni	Cu	B	Ca	K	Mg	Na	P	S	Si	Sr
Unit	mg O_2_/L	mg O_2_/L	mg/L	mg/L	mg/L	mg/L	mg/L	mg/L	mg/L	mg/L	mg/L	mg/L	mg/L	mg/L	mg/L
Value	92	-	50	4.64	0.27	1.54	0.12	81	79.8	67.2	259	0.36	58.3	9.7	0.97

^1^ COD—Chemical Oxygen Demand; ^2^ BOD—Biological Oxygen Demand.

**Table 3:** Characterization of synthetic tannery effluent.

Parameter	T	pH	Conductivity	Cl^−^	NH_4_^+^	Cr^3+^	Ca^2+^	K^+^	Mg^2+^	Na^+^	SO_4_^2−^
Unit	°C	-	mS/cm	mg/L	mg/L	mg/L	mg/L	mg/L	mg/L	mg/L	mg/L
Value	20	3.6	2.8	746.1	48.4	50	81	79.8	67.2	259	312.3

**Table 4:** Characteristics of the nanofiltration (NF) and reverse osmosis (RO) membranes studied, according to the manufacturers’ data.

Membrane	Max. Temperature (°C)	Max. Pressure (bar)	Salt Rejection (%)
NF270	45	35	80 (NaCl), 50 (CaCl_2_), 99.3 (MgSO_4_)
NF90	35–45	41	90–96 (NaCl)
BW30	45	41	99.4 (NaCl), 99.4 (CaCl_2_), 99.7 (MgSO_4_)
SW30	45	69	99.4 (NaCl)

**Table 5:** Pure water membrane permeabilities for the studied RO and NF membranes.

Membrane	Pure Water Permeability L/(h m^2^ bar)		Degree of Recovery (%)
	Before	After	
NF270 (pH 1.2)	7.729	7.353	95.135
NF 270 (pH 2.2)	8.594	7.263	84.512
NF270 (pH 3.6)	8.325	8.241	98.991
NF270 (pH 6.1)	8.858	7.218	81.486
NF90 (pH 1.2)	5.906	1.524	25.804
NF90 (pH 2.2)	7.172	0.840	11.712
NF90 (pH 3.6)	6.751	2.600	38.513
NF90 (pH 6.1)	7.320	3.971	54.249
BW30 (pH 1.2)	3.128	2.707	86.541
BW30 (pH 2.2)	3.280	2.884	87.927
BW30 (pH 3.6)	3.788	2.911	76.848
BW30(pH 6.1)	3.395	2.618	77.113
SW30 (pH 1.2)	0.893	0.33	36.954
SW30 (pH 2.2)	0.804	0.293	36.443
SW30 (pH 3.6)	0.809	0.485	59.951
SW30 (pH 6.1)	0.803	0.503	62.640
